# Supplementary material for: Role of tea plantations in the maintenance of bird diversity in Anji County, China
Source: PeerJ. 2023 Feb 13;11:e14801. doi: 10.7717/peerj.14801 (PMC9933740; doi:10.7717/peerj.14801)
Supplement: Supplemental Information 5 — Obs: observed I values for each variable; Z-scores are calculated as (observed - mean(randomizations))/sd(randomizations). [file peerj-11-14801-s005.docx]

| **Bird guild** | **Dependent variable** | **variable** | **Obs** | **Z.score** | **Sig95** |
| --- | --- | --- | --- | --- | --- |
| Completion | Richness | Plant | 0.97 | 0.70 |  |
|  |  | CONTAG | 2.79 | 3.61 | * |
|  |  | Quality | 2.95 | 3.65 | * |
|  | Abundance | Altitude | 0.89 | -0.34 |  |
|  |  | Plant | 1.09 | -0.29 |  |
|  |  | PD | 1.39 | -0.16 |  |
|  |  | LPI | 3.04 | 0.56 |  |
|  |  | Quality | 6.36 | 1.93 | * |
|  |  | North-South | 2.91 | 0.40 |  |
| Nature-Dependent | Richness | Plant | 1.03 | 1.36 |  |
|  |  | PD | 1.01 | 1.22 |  |
|  |  | CONTAG | 2.48 | 4.45 | * |
|  |  | Quality | 1.11 | 1.57 |  |
|  |  | Tea | 0.60 | 0.36 |  |
|  | Abundance | Plant | 2.32 | 1.98 | * |
|  |  | PD | 1.72 | 1.22 |  |
|  |  | CONTAG | 3.08 | 2.71 | * |
|  |  | Quality | 1.70 | 1.07 |  |
|  |  | Tea | 1.44 | 0.98 |  |
|  |  | Slope | 0.56 | -0.15 |  |
| Urban-Dependent | Richness | CONTAG | 1.26 | 1.55 |  |
|  |  | Quality | 4.33 | 7.34 | * |
|  | Abundance | Altitude | 2.04 | -0.05 |  |
|  |  | PD | 1.94 | -0.15 |  |
|  |  | LPI | 2.30 | -0.02 |  |
|  |  | Quality | 10.15 | 2.24 | * |
|  |  | Slope | 2.54 | 0.12 |  |
|  |  | North-South | 5.01 | 0.89 |  |
